# {*N*,*N*′-Bis[1-(2-pyrid­yl)ethyl­idene]ethane-1,2-diamine-κ^4^ *N*,*N*′,*N*′′,*N*′′′}bis­(thio­cyanato-κ*N*)manganese(II)

**DOI:** 10.1107/S1600536810021550

**Published:** 2010-06-16

**Authors:** Fu-Ming Wang

**Affiliations:** a Department of Chemistry, Dezhou University, Dezhou Shandong 253023, People’s Republic of China

## Abstract

The mol­ecule of the title compound, [Mn(NCS)_2_(C_16_H_18_N_4_)], has crystallographic twofold rotation symmetry, with the Mn^II^ atom lying on the rotation axis. The Mn^II^ atom is six-coordinated by four N atoms of the Schiff base ligand and by two N atoms of two thio­cyanate ligands, forming a distorted octa­hedral geometry.

## Related literature

For background to Schiff base compounds, see: Ruck & Jacobsen (2002 ▶); Mukhopadhyay *et al.* (2003 ▶); Polt *et al.* (2003 ▶); Mukherjee *et al.* (2001 ▶). For complexes derived from *N*,*N*′-bis­(1-(pyridin-2-yl)ethyl­idene)ethane-1,2-diamine, see: Gourbatsis *et al.* (1998 ▶); Louloudi *et al.* (1999 ▶); Karmakar *et al.* (2002 ▶); Banerjee *et al.* (2004 ▶). For related Mn^II^ complexes with Schiff bases, see: Louloudi *et al.* (1999 ▶); Sra *et al.* (2000 ▶); Karmakar *et al.* (2005 ▶); Deoghoria *et al.* (2005 ▶). For the synthesis of the Schiff base, see: Gourbatsis *et al.* (1990 ▶).
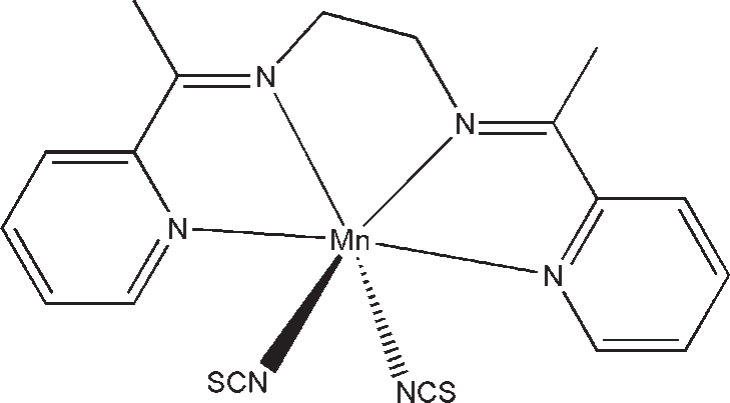


## Experimental

### 

#### Crystal data

[Mn(NCS)_2_(C_16_H_18_N_4_)]
*M* *_r_* = 437.44
Monoclinic, 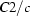

*a* = 12.570 (4) Å
*b* = 16.341 (5) Å
*c* = 9.962 (3) Å
β = 90.857 (4)°
*V* = 2045.9 (10) Å^3^
*Z* = 4
Mo *K*α radiation
μ = 0.86 mm^−1^
*T* = 298 K
0.17 × 0.15 × 0.15 mm

#### Data collection

Bruker SMART CCD area-detector diffractometer
Absorption correction: multi-scan (*SADABS*; Sheldrick, 1996 ▶) *T*_min_ = 0.867, *T*_max_ = 0.881
4479 measured reflections
2173 independent reflections
1590 reflections with *I* > 2σ(*I*)
*R*_int_ = 0.023

#### Refinement

*R*[*F* ^2^ > 2σ(*F* ^2^)] = 0.039
*wR*(*F* ^2^) = 0.093
*S* = 1.02
2173 reflections
124 parameters
H-atom parameters constrained
Δρ_max_ = 0.26 e Å^−3^
Δρ_min_ = −0.35 e Å^−3^

### 

Data collection: *SMART* (Bruker, 1998 ▶); cell refinement: *SAINT* (Bruker, 1998 ▶); data reduction: *SAINT*; program(s) used to solve structure: *SHELXS97* (Sheldrick, 2008 ▶); program(s) used to refine structure: *SHELXL97* (Sheldrick, 2008 ▶); molecular graphics: *SHELXTL* (Sheldrick, 2008 ▶); software used to prepare material for publication: *SHELXTL*.

## Supplementary Material

Crystal structure: contains datablocks global, I. DOI: 10.1107/S1600536810021550/ci5097sup1.cif

Structure factors: contains datablocks I. DOI: 10.1107/S1600536810021550/ci5097Isup2.hkl

Additional supplementary materials:  crystallographic information; 3D view; checkCIF report

## Figures and Tables

**Table 1:** Selected bond lengths (Å)

Mn1—N3	2.127 (2)
Mn1—N2	2.263 (2)
Mn1—N1	2.376 (2)

**Fig. 1.**
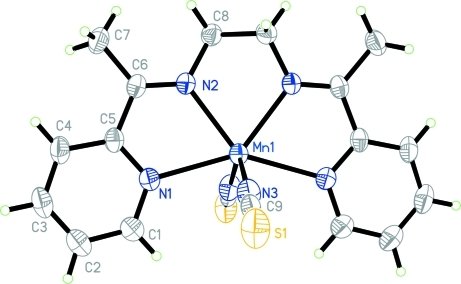
The molecular structure of the title compound, showing the atom-numbering scheme. Displacement ellipsoids are drawn at the 30% probability level. Unlabelled atoms are related to labelled atoms by the symmetry operation (1 - x, y, 1/2 - z).

## supplementary crystallographic information

### Comment

Metal complexes with Schiff bases have been known since 1840. The Schiff bases and their complexes have played an important role in the development of coordination chemistry, biological and material sciences (Ruck & Jacobsen, 2002; Mukhopadhyay *et al.*, 2003; Polt *et al.*, 2003; Mukherjee *et al.*, 2001). A few complexes derived from *N*,*N*'-bis(1-(pyridin-2-yl)ethylidene)ethane-1,2-diamine have been reported (Gourbatsis *et al.*, 1998; Louloudi *et al.*, 1999; Karmakar *et al.*, 2002; Banerjee *et al.*, 2004). In this paper, the title new Mn(II) complex is reported.

The title compound possesses a crystallographic twofold rotation axis symmetry, Fig. 1. The Mn^II^ atom is six-coordinated by four N atoms of the Schiff base ligand *N*,*N*'-bis(1-(pyridin-2-yl)ethylidene)ethane-1,2-diamine, and by two N atoms from two thiocyanate ligands, forming a distorted octahedral geometry. The coordinate bond lengths (Table 1) are comparable with those observed in other similar manganese(II) complexes with Schiff bases (Louloudi *et al.*, 1999; Sra *et al.*, 2000; Karmakar *et al.*, 2005; Deoghoria *et al.*, 2005).

### Experimental

The Schiff base ligand *N*,*N*'-bis(1-(pyridin-2-yl)ethylidene)ethane-1,2-diamine was synthesized according to the literature method (Gourbatsis *et al.*, 1990). To a stirred methanol solution of the Schiff base ligand (1.0 mmol, 0.266 g) was added a methanol solution of manganese acetate (1.0 mmol, 0.245 g) and ammonium thiocyanate (1.0 mmol, 0.076 g). The mixture was boiled under reflux for 2 h, then cooled to room temperature. Brown block-like single crystals, suitable for X-ray diffraction, were formed after slow evaporation of the solution in air for a few days.

### Refinement

H atoms were placed in geometrically idealized positions and constrained to ride on their parent atoms, with C–H distances of 0.93–0.97 Å, and with *U*_iso_(H) set at 1.2*U*_eq_(C) and 1.5*U*_eq_(C_methyl_).

### Crystal data

**Table:**

[Mn(NCS)_2_(C_16_H_18_N_4_)]	*F*(000) = 900
*M**_r_* = 437.44	*D*_x_ = 1.420 Mg m^−^^3^
Monoclinic, *C*2/*c*	Mo *K*α radiation, λ = 0.71073 Å
Hall symbol: -C 2yc	Cell parameters from 1222 reflections
*a* = 12.570 (4) Å	θ = 2.6–25.3°
*b* = 16.341 (5) Å	µ = 0.86 mm^−^^1^
*c* = 9.962 (3) Å	*T* = 298 K
β = 90.857 (4)°	Block, brown
*V* = 2045.9 (10) Å^3^	0.17 × 0.15 × 0.15 mm
*Z* = 4	

### Data collection

**Table:**

Bruker SMART CCD area-detector diffractometer	2173 independent reflections
Radiation source: fine-focus sealed tube	1590 reflections with *I* > 2σ(*I*)
graphite	*R*_int_ = 0.023
ω scan	θ_max_ = 27.0°, θ_min_ = 2.9°
Absorption correction: multi-scan (*SADABS*; Sheldrick, 1996)	*h* = −16→12
*T*_min_ = 0.867, *T*_max_ = 0.881	*k* = −20→20
4479 measured reflections	*l* = −12→10

### Refinement

**Table:**

Refinement on *F*^2^	Primary atom site location: structure-invariant direct methods
Least-squares matrix: full	Secondary atom site location: difference Fourier map
*R*[*F*^2^ > 2σ(*F*^2^)] = 0.039	Hydrogen site location: inferred from neighbouring sites
*wR*(*F*^2^) = 0.093	H-atom parameters constrained
*S* = 1.02	*w* = 1/[σ^2^(*F*_o_^2^) + (0.0354*P*)^2^ + 1.1621*P*] where *P* = (*F*_o_^2^ + 2*F*_c_^2^)/3
2173 reflections	(Δ/σ)_max_ = 0.001
124 parameters	Δρ_max_ = 0.26 e Å^−^^3^
0 restraints	Δρ_min_ = −0.35 e Å^−^^3^

### Special details

**Table:**

Geometry. All e.s.d.'s (except the e.s.d. in the dihedral angle between two l.s. planes) are estimated using the full covariance matrix. The cell e.s.d.'s are taken into account individually in the estimation of e.s.d.'s in distances, angles and torsion angles; correlations between e.s.d.'s in cell parameters are only used when they are defined by crystal symmetry. An approximate (isotropic) treatment of cell e.s.d.'s is used for estimating e.s.d.'s involving l.s. planes.
Refinement. Refinement of *F*^2^ against ALL reflections. The weighted *R*-factor *wR* and goodness of fit *S* are based on *F*^2^, conventional *R*-factors *R* are based on *F*, with *F* set to zero for negative *F*^2^. The threshold expression of *F*^2^ > σ(*F*^2^) is used only for calculating *R*-factors(gt) *etc*. and is not relevant to the choice of reflections for refinement. *R*-factors based on *F*^2^ are statistically about twice as large as those based on *F*, and *R*- factors based on ALL data will be even larger.

### Fractional atomic coordinates and isotropic or equivalent isotropic displacement parameters (Å^2^)

**Table:**

	*x*	*y*	*z*	*U*_iso_*/*U*_eq_	
Mn1	0.5000	0.28864 (3)	0.2500	0.04525 (18)	
N1	0.42596 (16)	0.32743 (14)	0.03855 (19)	0.0533 (5)	
N2	0.44344 (15)	0.17790 (12)	0.13277 (18)	0.0471 (5)	
N3	0.63772 (18)	0.34541 (15)	0.1706 (2)	0.0648 (6)	
S1	0.82249 (6)	0.38624 (6)	0.04009 (8)	0.0872 (3)	
C1	0.4168 (2)	0.40396 (19)	−0.0058 (3)	0.0700 (8)	
H1	0.4354	0.4463	0.0523	0.084*	
C2	0.3812 (2)	0.4238 (2)	−0.1337 (3)	0.0770 (9)	
H2	0.3750	0.4781	−0.1605	0.092*	
C3	0.3554 (2)	0.3616 (2)	−0.2193 (3)	0.0774 (9)	
H3	0.3325	0.3729	−0.3065	0.093*	
C4	0.3636 (2)	0.28185 (19)	−0.1753 (3)	0.0658 (8)	
H4	0.3455	0.2388	−0.2323	0.079*	
C5	0.39910 (18)	0.26645 (16)	−0.0454 (2)	0.0492 (6)	
C6	0.40708 (18)	0.18264 (16)	0.0130 (2)	0.0482 (6)	
C7	0.3715 (2)	0.11213 (18)	−0.0729 (3)	0.0731 (8)	
H7A	0.4227	0.1028	−0.1418	0.110*	
H7B	0.3036	0.1246	−0.1134	0.110*	
H7C	0.3654	0.0639	−0.0184	0.110*	
C8	0.4512 (2)	0.10020 (15)	0.2047 (2)	0.0563 (7)	
H8A	0.4556	0.0555	0.1410	0.068*	
H8B	0.3881	0.0922	0.2580	0.068*	
C9	0.7137 (2)	0.36210 (16)	0.1152 (2)	0.0515 (6)	

### Atomic displacement parameters (Å^2^)

**Table:**

	*U*^11^	*U*^22^	*U*^33^	*U*^12^	*U*^13^	*U*^23^
Mn1	0.0431 (3)	0.0515 (3)	0.0411 (3)	0.000	0.0002 (2)	0.000
N1	0.0524 (12)	0.0574 (14)	0.0500 (12)	0.0008 (10)	−0.0029 (9)	0.0033 (10)
N2	0.0468 (11)	0.0525 (13)	0.0418 (11)	0.0001 (9)	−0.0008 (9)	−0.0012 (9)
N3	0.0507 (13)	0.0863 (17)	0.0575 (13)	−0.0072 (12)	0.0016 (10)	0.0114 (12)
S1	0.0612 (5)	0.1388 (9)	0.0619 (5)	−0.0238 (5)	0.0102 (4)	0.0159 (5)
C1	0.081 (2)	0.0640 (19)	0.0650 (17)	0.0042 (16)	−0.0056 (15)	0.0044 (15)
C2	0.080 (2)	0.078 (2)	0.0727 (19)	0.0090 (17)	−0.0060 (17)	0.0206 (17)
C3	0.069 (2)	0.104 (3)	0.0589 (17)	−0.0006 (18)	−0.0151 (15)	0.0212 (18)
C4	0.0610 (17)	0.084 (2)	0.0523 (15)	−0.0075 (15)	−0.0149 (12)	0.0052 (15)
C5	0.0352 (12)	0.0679 (17)	0.0444 (13)	−0.0030 (11)	−0.0011 (10)	0.0014 (12)
C6	0.0387 (13)	0.0629 (16)	0.0430 (13)	−0.0037 (11)	0.0018 (10)	−0.0049 (11)
C7	0.086 (2)	0.077 (2)	0.0553 (16)	−0.0141 (17)	−0.0139 (15)	−0.0073 (14)
C8	0.0657 (17)	0.0558 (16)	0.0475 (14)	−0.0064 (13)	−0.0019 (11)	0.0004 (11)
C9	0.0520 (15)	0.0619 (17)	0.0405 (12)	−0.0004 (13)	−0.0061 (11)	0.0071 (11)

### Geometric parameters (Å, °)

**Table:**

Mn1—N3	2.127 (2)	C2—C3	1.363 (4)
Mn1—N3^i^	2.127 (2)	C2—H2	0.93
Mn1—N2	2.263 (2)	C3—C4	1.378 (4)
Mn1—N2^i^	2.263 (2)	C3—H3	0.93
Mn1—N1^i^	2.376 (2)	C4—C5	1.386 (3)
Mn1—N1	2.376 (2)	C4—H4	0.93
N1—C1	1.331 (3)	C5—C6	1.491 (4)
N1—C5	1.341 (3)	C6—C7	1.500 (3)
N2—C6	1.273 (3)	C7—H7A	0.96
N2—C8	1.461 (3)	C7—H7B	0.96
N3—C9	1.144 (3)	C7—H7C	0.96
S1—C9	1.617 (3)	C8—C8^i^	1.512 (5)
C1—C2	1.382 (4)	C8—H8A	0.97
C1—H1	0.93	C8—H8B	0.97
			
N3—Mn1—N3^i^	128.28 (13)	C1—C2—H2	120.9
N3—Mn1—N2	114.09 (8)	C2—C3—C4	119.4 (3)
N3^i^—Mn1—N2	106.83 (8)	C2—C3—H3	120.3
N3—Mn1—N2^i^	106.83 (8)	C4—C3—H3	120.3
N3^i^—Mn1—N2^i^	114.09 (8)	C3—C4—C5	119.3 (3)
N2—Mn1—N2^i^	73.78 (10)	C3—C4—H4	120.3
N3—Mn1—N1^i^	84.43 (8)	C5—C4—H4	120.3
N3^i^—Mn1—N1^i^	82.20 (8)	N1—C5—C4	121.5 (2)
N2—Mn1—N1^i^	141.89 (7)	N1—C5—C6	115.1 (2)
N2^i^—Mn1—N1^i^	68.89 (7)	C4—C5—C6	123.4 (2)
N3—Mn1—N1	82.20 (8)	N2—C6—C5	116.3 (2)
N3^i^—Mn1—N1	84.43 (8)	N2—C6—C7	126.0 (2)
N2—Mn1—N1	68.89 (7)	C5—C6—C7	117.7 (2)
N2^i^—Mn1—N1	141.89 (7)	C6—C7—H7A	109.5
N1^i^—Mn1—N1	149.06 (11)	C6—C7—H7B	109.5
C1—N1—C5	118.1 (2)	H7A—C7—H7B	109.5
C1—N1—Mn1	125.15 (18)	C6—C7—H7C	109.5
C5—N1—Mn1	116.51 (16)	H7A—C7—H7C	109.5
C6—N2—C8	122.2 (2)	H7B—C7—H7C	109.5
C6—N2—Mn1	122.72 (17)	N2—C8—C8^i^	109.89 (15)
C8—N2—Mn1	115.07 (14)	N2—C8—H8A	109.7
C9—N3—Mn1	166.9 (2)	C8^i^—C8—H8A	109.7
N1—C1—C2	123.5 (3)	N2—C8—H8B	109.7
N1—C1—H1	118.3	C8^i^—C8—H8B	109.7
C2—C1—H1	118.3	H8A—C8—H8B	108.2
C3—C2—C1	118.2 (3)	N3—C9—S1	178.7 (2)
C3—C2—H2	120.9		

Symmetry codes: (i) −*x*+1, *y*, −*z*+1/2.
